# Ninety-one years of midwifery continuity of care in low and middle-income countries: a scoping review

**DOI:** 10.1186/s12913-025-12612-0

**Published:** 2025-03-28

**Authors:** Qorinah Estiningtyas Sakilah Adnani, Ela Nurfitriyani, Yunri Merida, Siti Khuzaiyah, Giyawati Yulilania Okinarum, Ari Indra Susanti, Victor Abiola Adepoju, Sarena Haji Hashim

**Affiliations:** 1 Department of Public Health, Faculty of Medicine, Universitas Padjadjaran, Bandung, Indonesia; 2 Master of Midwifery Program, Faculty of Medicine, Universitas Padjadjaran, Bandung, Indonesia; 3 Midwifery Program, Guna Bangsa Health Sciences School, Yogyakarta, Indonesia; 4 Midwifery Program, Faculty of Health Science, Universitas Muhammadiyah Pekajangan, Pekalongan, Indonesia; 5 PAP Rashidah Sa’adatul Bolkiah, Institute of Health Sciences, Universiti Brunei Darussalam, Bandar Seri Begawan, Brunei Darussalam; 6 Professional Midwives Program, Faculty of Health Science, Universitas Respati Yogyakarta, Yogyakarta, Indonesia; 7 Department of HIV and Infectious Diseases, Jhpiego (an Affiliate of John Hopkins University), Abuja, Nigeria; 8 School of Nursing and Midwifery, La Trobe University, Melbourne, VIC Australia

**Keywords:** Continuity of midwifery care, Developing countries, Maternal health, Midwifery

## Abstract

**Background:**

Midwifery continuity of care during pregnancy, childbirth, and postpartum is essential for improving maternal and neonatal health outcomes. In low- and middle-income countries (LMICs), however, challenges such as healthcare worker shortages, limited infrastructure, poor healthcare access, and cultural barriers often hinder the effective provision of midwifery services. These issues contribute to unsustainable and inadequate care, adversely affecting maternal and newborn health. This study examines the impact of these challenges on the midwifery continuity of care and its subsequent effect on maternal and neonatal outcomes.

**Methods:**

A scoping review was conducted following Arksey and O’Malley’s framework. We analyzed 43 articles published between 1932 and 2023 across four databases. Included studies were conducted in LMICs, focused on continuous care models, and published in English. The review aimed to capture the varied impacts of midwifery care on health outcomes.

**Results:**

The review found that midwifery continuity of care in LMICs significantly improves maternal and newborn health by reducing medical interventions, increasing physiological births, and enhancing maternal satisfaction and breastfeeding rates. The approach also lowers newborn mortality and morbidity. Success factors include community acceptance, midwives’ cultural competence, and collaboration with traditional birth attendants. Barriers such as insufficient funding and resistance to change persist. Midwife-led continuity of care (MLCC) was associated with a 16% reduction in neonatal loss and a 24% reduction in pre-term births. Also, MLCC decreases newborn mortality by 10–20% and increases breastfeeding rates by up to 30%. Effective implementation requires integrating midwifery services into existing health systems, securing funding, expanding training, and strengthening community partnerships.

**Conclusions:**

Midwifery continuity of care enhances maternal and neonatal health in LMICs by minimizing unnecessary medical interventions and improving maternal satisfaction and breastfeeding outcomes. However, cultural and socioeconomic factors influence its acceptance. Further research is needed to integrate traditional birth attendants into formal health systems, overcome resistance to change, and develop strategies for effective collaboration between traditional and professional care providers.

**Supplementary Information:**

The online version contains supplementary material available at 10.1186/s12913-025-12612-0.

## Background

The midwifery profession promotes women-centred care and normal physiological childbirth as a vital part of the maternal healthcare system. Midwives are highly trained healthcare professionals who provide a continuum of care to women during pregnancy, birth, and postpartum period. Continuity of care involves a dedicated midwife or a small team providing consistent and comprehensive support, ensuring personalized attention throughout pregnancy, childbirth, and postpartum. This strategy differs from fragmented and episodic care, in which women may receive care from multiple providers at different phases, often resulting in disjointed and unsatisfactory outcomes. High-quality midwifery care can significantly decrease maternal and neonatal mortality and morbidity in high, low, and lower-middle-income nations. It must be supported by efficient education [1].

Midwifery continuity of care has emerged as a key and promising maternal and newborn healthcare approach. Quality midwifery care during pregnancy, labour, and postpartum is critical for mothers and neonatal health outcomes [2]. Based on the current evidence in high-income countries, implementing midwifery continuity of care has improved maternal and neonatal health outcomes. Continuity of care has been linked to lower rates of interventions such as cesarean sections, episiotomies, and instrumental births, enhanced maternal satisfaction, and an increased likelihood of commencing and maintaining breastfeeding [3]. However, in many low and middle-income countries (LMICs), births and deaths have a significant impact on women, their families, and health services. Low resources, insufficient infrastructure, and unequal access to healthcare services can also promote sub-optimal maternal and newborn outcomes [2] There is an interest to investigate the potential benefits of midwifery continuity of care in LMICs, where maternal and newborn death rates remain unacceptably high [3].

Despite the evidence of midwifery continuity of care as a viable method, little is known about its implementation and effectiveness in LMICs. These countries frequently confront unique problems, such as underdeveloped health systems, insufficient infrastructure, cultural beliefs and practices, socioeconomic inequities, and geographical constraints, which can impact the feasibility and outcomes of continuity-of-care approaches. The evidence basis for midwifery continuity of care in LMICs is relatively underreported. Available studies are fragmented, inconclusive, and lack detailed synthesis.

This scoping review aims to provide an updated overview of midwifery continuity of care in LMICs. It offers insights into its potential to improve maternal and newborn health outcomes in these contexts. By addressing these questions, the review will inform policy and practice, guide future research, and contribute to strengthening maternity and newborn healthcare systems in resource-constrained settings. Understanding the role of midwifery continuity of care is essential as the global community works towards achieving the Sustainable Development Goals (SDGs), particularly those focused on maternal and child health.

## Methods

Scoping reviews are an increasingly popular method for evaluating evidence in health research. This study utilized the scoping review methodology proposed by Arksey and O’Malley [4] As delineated by this framework, there are six distinct stages: (1) formulation of the research question; (2) identification of related studies; (3) study selection; (4) data collection; and (5) compilation, summarization, and reporting of findings.

### Stage 1. Formulation of the research question

This review paper aims to answer the following essential questions by methodically examining and summarising the current evidence: What is the current knowledge about implementing midwifery continuity of care strategies in LMICs? What are the lived experiences among women and families in LMICs linked with midwifery continuity of care? What barriers and enablers exist to adopting midwifery continuity of care in LMICs? How do contextual factors such as cultural beliefs, socioeconomic status, and health system capacity affect the effectiveness of midwifery continuity of care models in LMICs?

### Stage 2. Identification of related studies

The investigators searched the title and abstract of peer-reviewed and grey literature published since 1 January 1932 using synonyms and MeSH terms. We chose 1 January 1932 as the starting date because, in the early 1930s, most practicing midwives were based in LMICs and attended about half of all births. A comprehensive search was conducted across multiple databases, including PubMed, Embase, Scopus, and Web of Science, utilizing carefully selected keywords and Medical Subject Headings (MeSH) terms. Although the Maternal and Infant Care database is highly relevant to our topic, we were unable to include it in our search as none of the authors had institutional access. To capture relevant grey literature, we also included sources such as the Partnership for Maternal, Newborn, and Child Health, the Maternal Health Task Force, the Healthy Newborn Network, UNICEF, and the World Health Organization. A total of 3,408 unique publications were identified in the initial search that met the criteria for screening based on their title and abstracts. Furthermore, in addition to the articles already included, we examined the reference lists of the reviews identified during the title and abstract screening.

We applied keyword searches with filters to refine the results based on a predetermined framework. The chosen keywords were: (continuity) AND (midwifery care) AND (low middle-income countries). To ensure a systematic selection process, the authors established the inclusion and exclusion criteria outlined in Table 1.

**Table 1:** Inclusion and exclusion criteria

Inclusion criteria	Exclusion criteria
Article published from 1 January 1932 – December 2023 Full-text articles (Open access)	Editorial, conference, abstracts, newspaper articles, and non-full text articles.
Articles are written in English.	Studies show there is no correlation with midwifery continuity of care in LMICs
Randomised controlled trials, cohort studies, cross-sectional studies, and qualitative research	Articles do not focus on the current continuity of care in LMICs.
Articles addressing aspects of implementing midwifery continuity of care strategies in LMICs; live experiences among women and families in LMICs linked with midwifery continuity of care; barriers and enablers to adopting midwifery continuity of care in LMICs; and contextual factors such as cultural beliefs, socioeconomic status, and health system capacity, as well as the impact of midwifery continuity of care models in LMICs	
Studies related to midwifery care focusing on pregnant women, childbirth, and the postpartum period	

### Stage 3. Study selection

During the preliminary search, 3,408 articles were identified and archived in the Zotero bibliography management system. We then applied our framework to exclude articles that did not meet the inclusion criteria. The results were documented, including the number of articles retained. A PRISMA flow diagram was created to illustrate the screening process.

After removing 1,841 duplicate records, largely due to articles appearing in multiple databases, 1,567 papers remained. Subsequently, 1,003 publications were excluded based on irrelevant titles and abstracts. Two researchers with relevant expertise and shared research interests further assessed 157 publications. The evaluation focused on studies that provided key insights into the implementation of continuity of care in LMICs, the lived experiences of women and families, contextual factors such as cultural beliefs, socioeconomic status, and health system capacity, as well as the impact of midwifery continuity of care models in LMICs.

**Fig. 1:**
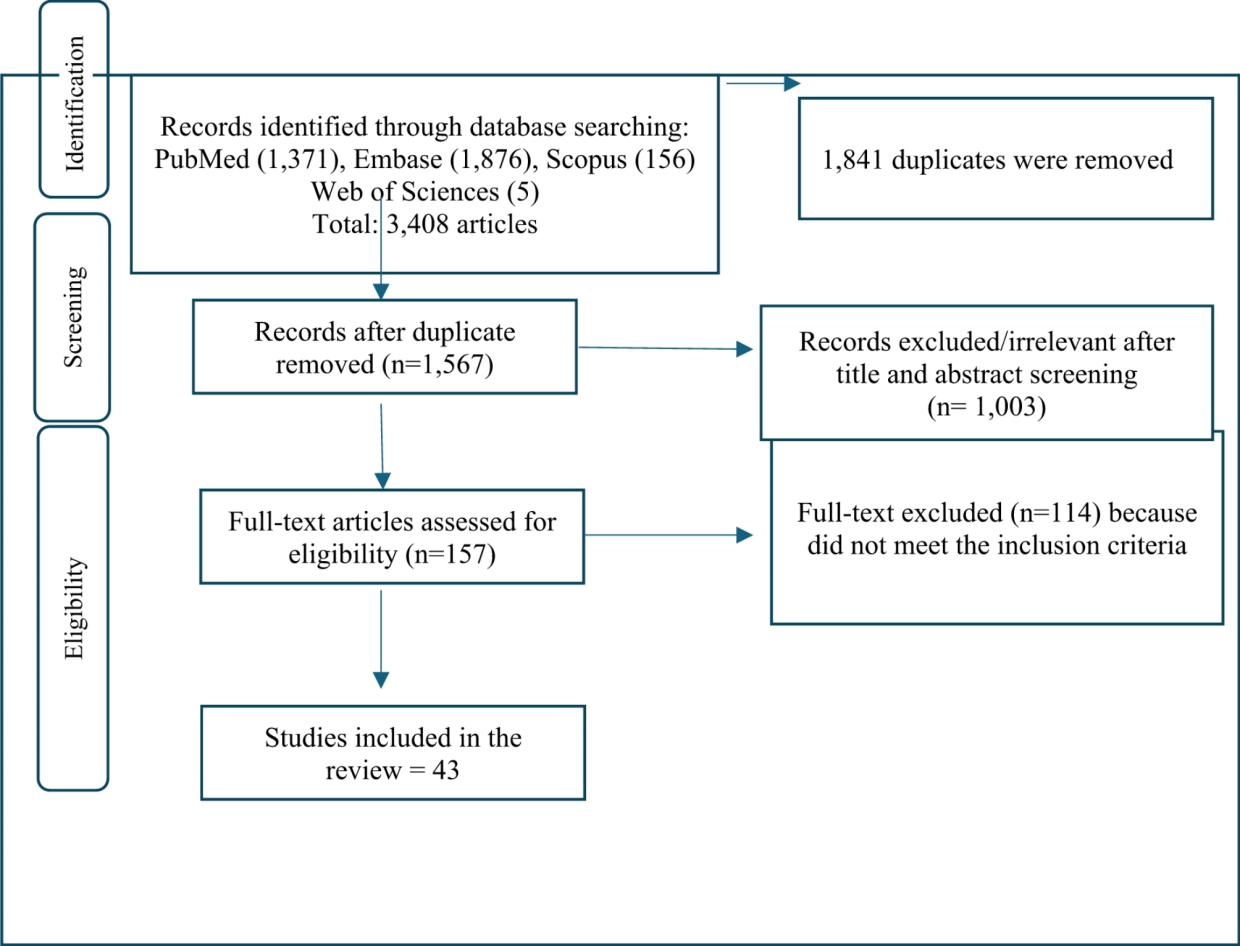
PRISMA flow diagram for scoping review process

Ultimately, 114 publications were excluded for not meeting the inclusion criteria. A total of 43 papers were finally included in the study. Figure 1 illustrates the screening process and its outcomes.

### Stage 4. Data collection

Two researchers initially refined the search results by screening titles and abstracts before conducting a full-text assessment of potentially relevant papers. A total of 43 articles were selected, and their key results were documented. To systematically gather data, we used a standardized extraction form that included each study’s title, authors, year, country, objectives, design, population, and main findings. The extracted data also included findings on midwifery continuity of care models, measured outcomes, and reported maternal and neonatal health results.

Qualitative studies were further categorized based on factors that facilitated or hindered midwifery continuity of care. Two researchers independently conducted data extraction, and any discrepancies were resolved by a third reviewer.

### Stage 5. Compilation, summarization, and reporting of findings

Following the selection of 43 final studies (Fig. 1), appropriate methods were used to assess the methodological rigor of each included study, as outlined in Table 2. For quantitative research involving analytical cross-sectional studies, cohort studies, and randomized controlled trials, we applied the Joanna Briggs Institute (JBI) Critical Appraisal Checklists. For qualitative studies, we used the JBI Critical Appraisal Checklist for Qualitative Research, and for scoping reviews and research syntheses, we employed the corresponding JBI checklist.

**Table 2:** Summary of key findings

No	Countries	Study Aim	Study Design, Population	Results or Key Findings
A1	Countries: Afrika Selatan, Uganda, Kenya, Tanzania, Zimbabwe, Malawi, Zambia, India, Brazil, Malaysia, Iran, and Bangladesh [5]	This study aims to delineate the objectives of strategies employed by low- and middle-income countries (LMICs) in creating palliative care services in rural areas.	The review approach entailed doing a thorough search in four electronic databases (Ovid MEDLINE, Ovid Emcare, Embase classic + Embase, and CINAHL), which led to the inclusion of 7 out of 30 publications. The assessed papers were determined to have a low methodological quality.	Healthcare providers and volunteers were given training and mentorship. Effective palliative care in rural regions of low- and middle-income nations relies on cooperation among healthcare practitioners, volunteers, religious authorities, and technology.
A2	Countries: Guatemala [6]	To evaluate the existing collaboration with traditional birth attendants who have access to mHealth technology to improve the detection of high-risk pregnancies and childbirth difficulties.	The study encompassed a population of 95,000 traditional birth attendants (TBA), with a subset of 41 TBAs being used as a sample. The data were analysed using statistical process control techniques.	During 12 months, traditional birth attendants (TBAs) assisted in 847 births, while referral rates increased from 14 to 27.5. The implementation of Obstetric Care Navigation (OCN) significantly improved the quality of treatment at the facility level, with an increase from 24–62%. The most frequent causes for referrals were hypertensive problems and protracted labour. OCN also offered emergency transportation and labour assistance, showcasing its efficacy as a practical and patient-focused improvement for maternity care.
A3	The countries that are the focus of this study include Madagascar, Somalia, Ethiopia, Chad, Niger, Sudan, Sierra Leone, Burundi, Uganda, Nigeria, Liberia, India, Kenya, Rwanda, Zambia, Tanzania, South Africa, Botswana, Morocco, Ghana, Uzbekistan, Vietnam, Indonesia, Pakistan, and Cambodia. Additionally, the research also encompasses countries such as Bangladesh, Nepal, Guinea, Mali, Mozambique, and Senegal [7].	The objective is to assess the impact and expenses and create novel simulations addressing the prospective outcomes for mothers, fetuses, and infants. This will be done by improving midwifery services, including family planning, in 58 countries with low and middle incomes.	The research identified precise interventions involving midwives or obstetricians, and the sample included 58 low- and middle-income nations.	While obstetricians are more effective in reducing maternal and fetal fatalities, expanding midwifery services can avoid a greater number of newborn deaths compared to exclusively focusing on obstetrics. Midwifery presents a financially efficient approach to reducing mortality rates by delivering comprehensive care from the home to the medical facility. In addition, midwives excel in providing breastfeeding counselling, resulting in more substantial decreases in infant mortality compared to obstetricians.
A4	Countries: India, Ethiopia, Ghana, Uganda, Kenya, Nepal, Tanzania, Nigeria, Gambia, Malawi, Thailand, Liberia, Kamboja, Laos, dan Rwanda [8].	The purpose is to present an outline of the difficulties associated with human resources for health in guaranteeing high-quality care for newborns in countries with low and intermediate incomes.	A scoping thematic analysis was performed, utilising papers obtained through database searches and manual scrutiny of references and country reports. Thematic analysis was subsequently used to identify and classify ten distinct HRH issues.	There is a notable deficiency in healthcare professionals that possess expertise in neonatal care. Examining this problem provides valuable perspectives for formulating new WHO approaches to address these difficulties and enhance the workforce for neonatal care, ultimately resulting in improved outcomes for newborns.
A5	Country: Zambia [9]	The objective is to evaluate the effects of midwife training and infant care on decreasing neonatal and perinatal mortality rates in developing nations.	The design included a Train-the-Trainer model, which included a group of midwives and 71,689 neonates.	The perinatal mortality rates decreased, while the stillbirth rates remained constant. There was a considerable drop in the mortality rate of newborns within the first seven days after receiving training in the Neonatal Resuscitation Program. Providing training to midwives in Essential Newborn Care (ENC) resulted in a decrease in 7-day infant mortality in low-risk clinics. Further enhancing the death rates could be achieved by providing further foundational instruction in neonatal resuscitation.
A6	Country: Bangladesh [10]	To examine equity in utilising home-based versus facility-based obstetric services in rural Bangladesh through an observational study.	A multinomial logistic regression and a binomial log-link regression analysis were conducted.	This study examined the disparities between home-based and facility-based obstetric services in rural Bangladesh. Findings revealed that despite the expansion of facility-based care, home births attended by midwives remained predominant. The study emphasizes the need to evaluate the cost, feasibility, and effectiveness of these approaches to improve maternal health equity.”
A7	Countries: Zambia, India, Kenya [11]	To explore how, why, to what extent, and for whom Community Engagement (CE) influences both intended and unintended outcomes in Maternal and Newborn Health (MNH) programming, emphasising the communication aspects of CE.	The realist review methodology focused on a sample of CE interventions within Maternal and Newborn Health (MNH) programming.	A previous realist review on Community Engagement (CE) in health research highlighted the significance of fostering strong “working relationships” between communities and researchers. It also examined the key factors and processes influencing CE and its impact on the research process.
A8	Countries: Argentina, Kuba, Arab Saudi, Thailand, and Zimbabwe [12]	To compare the outcomes of antenatal care programs with reduced visits for low-risk women to those of standard care.	The trials conducted by the group involved a population of 60,000 women, with the sample consisting of pregnant and postpartum women.	Women expressed dissatisfaction with the fewer visits, feeling that the intervals were too extended. Although fewer visits might reduce costs, women in high-income countries typically had between 8.2 and 12 visits. In contrast, in low- and middle-income countries, many women had fewer than five visits, often with modifications to the content of each visit.
A9	Countries: Ghana, Uganda, Afrika Selatan, Indonesia, Afghanistan, Nepal, and India [13].	To create a comprehensive map of the literature on barriers to quality midwifery care using a systematic and replicable approach and validate the analytical framework developed during the Women Deliver session by thoroughly detailing the identified barriers and the reviewed literature types.	A systematic mapping was conducted, resulting in 9,126 items being retrieved across five databases and from a call for papers, which was narrowed down to 7,344 items. Data for analysis were extracted from all 82 selected items. The sample included midwives, maternity staff, nurse-midwives/nurses, obstetricians, paediatricians, physicians, and neonatal nurses.	The mapping findings reveal that quality midwifery care is impeded by socio-cultural, economic, and professional barriers, all of which are deeply rooted in gender inequality. The perception of midwifery as “women’s work” contributes to the “gender penalty,” positioning women at the lower end of occupational and economic hierarchies.
A10	Countries: Colombia [14]	To examine traditional birth attendants (TBAs) by exploring who they are, the number of births they attend globally, the locations where they provide delivery care, and their interactions with the formal healthcare system and the communities they serve.	A systematic mapping was conducted, focusing on a sample of pregnant women.	Although the role of Traditional Birth Attendants (TBAs) in healthcare is diminishing, they continue to be essential in rural areas where health services are limited. Despite efforts to enhance access to skilled healthcare providers in these regions, there remains a shortage of trained professionals and financial limitations.
A11	Countries: Kenya [15]	To create appropriate metrics for evaluating the quality of nursing care in low- and middle-income countries (LMICs) where such assessments are currently lacking.	Method: A literature review was conducted through a scoping review approach, utilising databases such as EMBASE, CINAHL, MEDLINE, and Google Scholar, with a sample of 52 studies focused on nursing care.	Kenya’s proposed Nursing Sensitive Indicators (NSIs) address a global need to monitor nursing care quality in low- and middle-income countries (LMICs). These indicators require validation and refinement to achieve standardisation, allowing LMICs to participate in or establish professional networks to enhance care quality.
A12	Countries: Ghana, Kenya, Malawi, Nigeria, Sierra Leone, Tanzania, Zimbabwe, Bangladesh, and Pakistan [16]	To identify the most efficient and effective methods for low- and middle-income countries (LMICs) to conduct pre-service and in-service education and training, ensuring that care providers are adequately prepared to deliver quality maternal and newborn care.	A Rapid Systematic Evidence Review was performed, involving a systematic search across databases including Medline, CINAHL, LILACs, PsycINFO, ERIC, and MIDIRs. The study focused on a population of 19, with a sample comprising maternal and newborn care providers.	The studies demonstrated improved knowledge and skills but lacked evidence regarding their impact and theoretical foundation. Aligning these skills with the Quality Maternal and Newborn Care framework is crucial for enhancing midwifery care and contributing to advancing the Sustainable Development Goals.
A13	Countries: Guatemala [17]	To investigate the availability of various providers who deliver care during pregnancy and to examine the specific characteristics of midwives.	The method was qualitative, with a sample focusing on pregnancy and the postpartum period.	In Guatemala, although 75% of midwives have received formal training and are encouraged to make referrals, the majority of pregnant women still do not seek care from biomedical providers.
A14	Countries: India, Sudan [18]	To explore community healthcare systems in developing countries.	The method used was qualitative, focusing on children under 5.	The community health center aims to deliver two-thirds of essential healthcare services, including prenatal supervision, midwifery, neonatal care, treatment of endemic diseases, and emergency care for accidents. Initial findings suggest that practical experience and community recognition play a more significant role in a health practitioner’s effectiveness than formal education. Additionally, enhancing diet, hygiene, and sanitation is crucial for optimizing community health outcomes.
A15	Countries: Somaliland, Kenya, Malawi, Swaziland (now known as Eswatini), Zimbabwe, Tanzania, and Sierra Leone [19].	Exploring ways to enhance critical obstetric and neonatal care in countries with limited resources.	The qualitative method involves 600 healthcare providers, including nurse-midwives, doctors, clinical officers, and specialists.	The training led to a significant increase in knowledge and skills (*p* < 0.001) in pregnancy and newborn care. Participants expressed high satisfaction levels, and the program promoted evidence-based practices and improved teamwork.
A16	Countries: Nigeria [20]	To observe the rise in maternal mortality rates in developing countries.	Quantitative method with a sample of pregnant individuals.	Global health policies should prioritise support for developing countries, emphasising financial and technological assistance. While universal formal education is a crucial strategy, challenges persist in modernising these societies.
A17	Countries: Nepal, India, Pakistan, Bangladesh, Tanzania, Uganda, Ethiopia, Senegal, Kenya, and Papua New Guinea [21]	To synthesise implementation lessons on birth kits, focusing on the context, user, usage requirements, and logistics of kit distribution.	A systematic literature review with a focus on maternal and child health. The review included 28 articles, which described a total of 21 birth kits used across 40 different countries.	The community health center aims to deliver two-thirds of essential healthcare services, including prenatal supervision, midwifery, neonatal care, treatment of endemic diseases, and emergency care for accidents. Initial findings suggest that practical experience and community recognition play a more significant role in a health practitioner’s effectiveness than formal education. Additionally, enhancing diet, hygiene, and sanitation is crucial for optimizing community health outcomes.
A18	Countries: Ghana [22]	To examine the coping strategies employed by Ghanaian midwives to manage and complete their work effectively.	This study utilised the Glaserian Grounded Theory. Data were gathered through non-participant observations and semi-structured interviews. The participants included 29 midwives working in labour/birthing environments, a pharmacist, a social worker, a National Health Insurance Scheme manager, and a health services manager.	The midwives’ motivation, driven by a deep commitment to safeguarding the lives of women and newborns and a strong passion for the midwifery profession, was identified as a key factor in overcoming workplace challenges. This dedication enabled them to adapt, take leadership in the birthing process and environment, and maintain engagement with professional and social networks, all of which contributed to their effectiveness in delivering quality care.
A19	Countries: Brazil [23]	Calculate the frequency of institutional delivery, SBA coverage, and the combination of delivery location and attendant type in low- and middle-income countries (LMICs). Similar assessments were recently released for 57 nations using DHS data, concentrating on the public and private sectors in four global areas.	Analyses Surveyed cross-sectional sample of 80 LMICs and women between the ages of 15 and 49 who are fertile	Comprehensive analyses that consider both the place of delivery and the type of birth attendant are essential for promoting safe childbirth, particularly in remote areas where skilled birth attendants are scarce. Home births attended by skilled birth attendants (SBAs) were more common among women in lower socioeconomic groups than in urban settings. Additionally, deliveries without the assistance of an SBA remain widespread in underserved and remote communities.
A20	Countries: Pakistan [24]	To gauge the Examination and evaluation of enhancements made to the high-quality healthcare systems in developing nations	We searched peer-reviewed databases MEDLINE, CINAHL, and PubMed for national and international literature.	Effective quality assessment methods can provide valuable guidance on optimizing resource allocation in developing countries, enabling healthcare systems to efficiently manage limited resources while addressing the needs of a growing population and improving healthcare services.
A21	Countries: Uganda [25]	Education aims to provide males with the necessary information and skills to support women throughout pregnancy and childbirth.	A scoping review was conducted using Bournemouth University’s iteration of the EBSCO Discovery Service (EDS) tool, identifying 33 studies. The findings highlight the rationale for involving men in maternity care.	Involving men in maternity care can significantly improve health outcomes for both mothers and infants. Through health education programs, men can acquire the knowledge and skills necessary to support women’s health during pregnancy, childbirth, and the postpartum period.
A22	Countries: Bangladesh, India, Kenya, Uganda, Zambia, Niger, Nepal, Cambodia, Senegal, Malawi, Papua N, Honduras, and Ukraine [3]	Examining cost-effective methods to boost the supply and use of maternity and newborn health care in low- and lower-middle-income nations	A systematic review was conducted using searches across six electronic bibliographic databases: Medline, Embase, Global Health, EconLit, Web of Science, and the NHS Economic Evaluation Database. The review included 48 publications focused on maternal and newborn health care.	It was determined that cost-saving measures could include establishing women’s support groups, utilizing community health workers and traditional birth attendants for newborn care at home, enhancing routine prenatal care services, implementing programs to ensure adherence to care standards, and promoting breastfeeding in maternity hospitals. However, comparing the cost-effectiveness of these various approaches proved challenging due to differences in metrics and evaluation methods.
A23	Countries: Nepal, India, Bangladesh, and Pakistan. [26]	To outline the elements influencing pregnant women’s care-seeking behaviour and investigate potential treatments that could increase the number of facility-based births among women in South Asia.	A literature review was conducted using various databases, including PubMed. Data sources from the World Health Organization (WHO), the United Nations Population Fund (UNFPA), and non-governmental organisations such as Safe Motherhood Nepal were also searched. The review included 100 articles.	A comprehensive approach is crucial to increasing the use of facility-based childbirth services among women in rural Nepal. This study highlights three key strategies that should be prioritized in South Asian countries to expand access to skilled birth attendants within healthcare institutions.
A24	Countries: Angola, Benin, Malawi, Mozambique, Nigeria, South Africa, Tanzania, Uganda, Senegal, and Zambia [27]	To investigate the delivery of midwife-led care in low- and middle-income nations.	A scoping review, an organised search of Pubmed, EMBASE (Ovid), Web of Science, Scopus, Google Scholar, The Cochrane Library, and a manual search of pertinent international organisation websites, journals, and grey literature were carried out. 3483 pieces of content	There is limited data on the effectiveness of midwife-led care in low- and middle-income countries, where it is not widely practiced. However, most studies have examined its implementation across different healthcare settings. The structure of midwife-led care varies considerably, along with differences in education, regulation, and training standards. While midwife-led care is available in many low- and middle-income countries, its quality is often restricted by inadequate support systems and resources.
A25	Countries: Iran [28]	To provide an overview of the qualitative research on the obstacles to PNC management in LMICs related to the healthcare system.	A systematic review of the qualitative literature was conducted by searching databases such as PubMed, Web of Knowledge, CINHAL, SCOPUS, Embase, and Science Direct for qualitative studies performed in low- and middle-income countries (LMICs). The sample population included 1,677 participants, comprising 629 pregnant women, 122 mothers, 240 healthcare providers, 54 key informants, 164 women of childbearing age, 380 community members, and 88 participants from other groups (such as male partners or other key informants).	The research emphasizes that postnatal care (PNC) in LMICs has substantial obstacles related to staffing, provision of services, availability, and infrastructure. Research undertaken in multiple countries with a range of participants, including healthcare workers, pregnant women, male partners, and community members, has shown an understanding of postnatal care (PNC) across the healthcare system.
A26	Countries Africa, Asia, and Latin America [29]	To examine the views, experiences, and behaviours of skilled birth attendants and their supporters; to identify the factors influencing intrapartum and postnatal care delivery in low- and middle-income countries; and to assess how these factors are represented in intervention studies.	Qualitative methods were used, involving doctors, midwives, nurses, auxiliary nurses, and their managers, with 31 studies included.	The effectiveness of care provided by skilled birth attendants (SBAs) is influenced by several aspects, including their training, supervision, staffing levels, remuneration, housing circumstances, and the presence of well-equipped facilities. Furthermore, the efficacy of collaboration, reliance, and communication among healthcare professionals and with mothers is crucial in ensuring the quality of care. SBAs have documented difficulties linked to each of these criteria.
A27	Countries: Afghanistan, Bangladesh, Brazil, Ecuador, Fiji, Gambia, Ghana, Guatemala, Guinea, Haiti, India, Indonesia, Iran, Malawi, Mexico, Morocco, Pakistan, Philippines, Sierra Leone, South Africa, Uganda, Yemen, Zambia, and Zimbabwe [30]	To determine which low- and middle-income countries (LMICs) have midwife-led birthing centres and to identify their key characteristics.	A scoping survey of professional midwives’ associations was conducted in Part 2, while a scoping examination of the peer-reviewed and grey literature was conducted in Part 1. A protocol wasn’t released before this work was done. Responses to the polls, which included a structured online questionnaire, came from 77 of the 137 low- and middle-income nations in the globe—nurses who work as midwives.	Midwife-led birthing centers were more prevalent in low- and lower-middle-income countries compared to upper-middle-income nations. The majority of these centers operated as independent midwife-led facilities. In middle-income countries, birthing centers were more commonly managed by public-sector midwives, whereas in low-income settings, midwives often operated independently. In some cases, a multidisciplinary team of healthcare professionals collaborated, while in others, midwives were the sole providers of care. However, challenges persisted in establishing effective referral networks and fully integrating the midwifery care model into existing healthcare systems.
A28	Countries: Ghana, India, Bangladesh, Pakistan, Uganda, Malawi, Kenya, Tanzania, Ethiopia, Nepal, and Honduras [31]	To assess how crucial infant care measures are implemented in LMICs	Systematic Reviews and Meta-analyses (PRISMA), 43 articles, population newborns based quantitative and qualitative study designs, and databases including MEDLINE, EMBASE, CINAHL, Cochrane Central, and the Global Health Library.	This study identifies several barriers to learning from the implementation of ENC in LMICs, specifically highlighting the insufficient description of interventions and implementation outcomes. To decrease the number of deaths among newborns, it is crucial to find ways to improve the reporting of implementation studies in this area, which ultimately lead to better service delivery and outcomes.
A29	Countries: Senegal, Burkina Faso, Mozambique, Tanzania, Rwanda, Mali, Guinea-Bissau, Vietnam, Bangladesh, Nigeria, Angola, Uganda, Gambia, India, and Indonesia [32]	To examine quantitative data regarding the impact of various birthing attendance techniques in low-income environments on maternal health	Design: Systematic review using quantitative methods. The databases searched included MEDLINE, EMBASE, Cochrane Library, BIOSIS Previews, Web of Science, CINAHL, and POPLINE, with 29 articles included in the review.	Comparing studies was challenging due to inconsistencies in defining key concepts such as “skilled birth attendance” and the tendency to evaluate multiple interventions without isolating the effects of individual components. However, some studies identified promising factors, including cost, accessibility, widespread availability of essential medications, and the need for specialized training. These findings highlight the importance of developing clear conceptual frameworks to assess both individual interventions and comprehensive care models effectively.
A30	Countries: Afghanistan, Bangladesh, Benin, Brazil, Cambodia, Ghana, Guatemala, Iran, Kenya, Laos, Malawi, Morocco, Mozambique, Nepal, Nigeria, Pakistan, Palestine, Peru, Rwanda, South Africa, Uganda, and Vietnam [33]	Combining data on implementation hurdles and enablers for midwife-led care for pregnant women in low- and middle-income countries (LMICs) from the viewpoints of care recipients, providers, and other interested parties.	A mixed-methods systematic review included 31 studies. Of these, ten studies involved all three groups, five focused on care providers and stakeholders, and one study concentrated on care recipients and care providers.	Women need to possess knowledge to properly utilise midwife-led care, while strong education and supervision are essential for midwives. Collaboration and reliable financial support are crucial, but political instability can present considerable obstacles to implementation in LMICs.
A31	Countries: Rwanda, Afghanistan, Jordan, and Botswana [34]	To assess the existing knowledge on mentorship for healthcare professionals to improve the quality of care in low- and middle-income countries (LMICs).	Scoping reviews were performed by conducting searches on OVID Medline, CINAHL, and EMBASE. The population comprised individuals from various healthcare professions, including nurses, community health workers, medical staff, doctors, physicians, rural health personnel, physician assistants, field workers, and clinical officers. The review encompassed a total of 78 papers.	Tailored mentorship programs created to target specific objectives and educational needs can potentially enhance the quality of care. Training programs that enhance the capabilities of individuals are both more enduring and more effective in promoting decentralised primary health care.
A32	Countries: Sub-Sahara Afrika, Asia Selatan [2]	To perform a qualitative meta-summary of the experiences of parents and healthcare professionals about post-stillbirth care in low- and middle-income countries (LMICs).	A systematic review and meta-summary were conducted, with searches performed in databases including AMED, EMBASE, MEDLINE, PsychINFO, BNI, and CINAHL. A total of 118 full-text articles were included in the review.	Women undergo a range of different and frequently unacknowledged sorrow after experiencing a stillbirth, which can result in being stigmatised and a decline in their social standing. Health systems that are well-developed and staffed with trained personnel can offer essential assistance and knowledge. Simultaneously, even crucial measures can greatly improve the experiences of women and their families during this challenging period.
A33	Countries: Ghana, Nigeria, Gambia, Burkina Faso, Ivory Coast, and Uganda [35]	The purpose of obstetric first aid in the community is to serve as a strategic component of the “Partners in Safe Motherhood” campaign to diminish maternal mortality rates.	Two complementary instructional sessions. Maternity care providers	The authors emphasize the critical importance of emergency care training for both healthcare professionals and paraprofessionals. They argue that such training should be complemented by educational and community mobilization efforts targeting families, local communities, and traditional birth attendants (TBAs). Establishing a shared understanding of the necessity and appropriate methods for timely intervention is essential to reducing maternal mortality.
A34	Countries India [36]	The objective is to examine the correlation between age groups and the utilisation of maternal healthcare services in India while also accounting for individual, household, and environmental factors.	The cohorts for age at childbirth are categorised as follows: 15–24 years old, 25–34 years old, and 35–49 years old pregnant woman	The findings emphasize the importance of age-sensitive interventions that tailor programs and incentives to meet women’s healthcare needs at different stages of their reproductive lives. Prioritizing the provision of essential maternal healthcare services is particularly crucial for illiterate women, those with limited autonomy, and individuals from disadvantaged socioeconomic backgrounds.
A35	Countries Malawi, Rwanda, Tanzania, Uganda, Mozambique and South Africa [37]	To gather qualitative research data on the views and experiences of community health workers (CHWs) about supervision in maternal and child health (MCH) programs in low- and middle-income countries (LMICs).	Qualitative Methods; 19 papers; databases: EMBASE, Medline, PsycINFO, ASSIA, ERIC, and CINAHL; patient and client experiences were excluded.	Engaging community health workers (CHWs) and supervisors in developing supervision models could offer benefits. Supportive supervision is crucial for sustaining the motivation and performance of Community Health Workers (CHWs). However, CHWs often see supervision as irregular and primarily centred on fault-finding rather than offering assistance. Supervisors must undergo training in supportive supervision techniques and be provided with sufficient time and resources to conduct effective supervision in the field.
A36	Countries: Fiji [38]	To investigate the strategies employed by nursing leaders and managers in a developing nation to impact patient safety.	Nursing leaders and managers were interviewed using semi-structured interviews.	The findings reflect the necessity of investigating the working conditions of front-line nurses, the direct correlation between improved nursing conditions and enhanced patient safety, the empowerment of nursing leaders and managers, and a stronger emphasis on patient-centred care. These insights can improve the global nursing community’s ability to provide more effective support to advance a patient safety agenda.
A37	Countries: Indonesia [39]	To delineate the evolution and advancement of the healthcare system in Indonesia.	Qualitative research	Indonesia’s healthcare system revolves around the pivotal community-based health service called Puskesmas. According to the World Health Organization, around 64% of fatalities in Indonesia are attributed to noncommunicable illnesses, which may be associated with the inadequately organised prehospital care system.
A38	Countries: Sri Lanka and Mongolia [40]	The objective is to assess the viewpoints of local healthcare providers regarding the key areas that need improvement in maternity and neonatal departments.	Qualitative studies on maternity and newborn healthcare	Health practitioners from high-resource countries placed greater emphasis on organizational structures compared to their counterparts in low-resource settings, focusing on clearly defining roles and responsibilities. While staff education is widely recognized as the most critical strategy for enhancing the quality of maternity and neonatal care, significant discrepancies in workplace objectives continue to exist.
A39	Countries: Tanzania [41]	To comprehensively describe the healthcare practitioners (HCPs) in Tanzania who are recognised as skilled birth attendants (SBAs). It also discusses the emergency obstetric care (EmOC) signal functions that these SBAs undertake and the problems they have in carrying out these functions. We conducted a cross-sectional study on healthcare professionals (HCPs) who provide maternity care services at eight health facilities in Moshi Urban District in northern Tanzania.	A total of 199 individuals participated in a Cross-sectional study conducted in various medical facilities.	Healthcare providers at all levels have identified two major challenges: inadequate supplies and equipment, and a lack of sufficient knowledge and skills in delivering Emergency Obstetric Care (EmOC). Discrepancies were observed between the performance of skilled birth attendants (SBAs) in medical facilities and the expectations set by the Ministry of Health and Social Welfare (MOHSW) regarding their EmOC responsibilities. Many key EmOC facilities were not functioning at full capacity, and only a limited number of medical practitioners were able to effectively perform all essential EmOC procedures. Enhancing working conditions and implementing competency-based in-service training programs for EmOC providers could significantly strengthen EmOC services in the district.
A40	Countries: Greece [42]	The objective is to gather data on how countries have improved their healthcare systems and deployed midwives in nations with high maternal mortality rates.	A qualitative study on the health of mothers and newborns.	In these countries, the efforts to enhance the health system have been characterised by expanding the network of healthcare facilities and increasing the number of women who give birth in these facilities. Additionally, there has been a focus on increasing the production of midwives, reducing financial obstacles, and a lesser emphasis on improving the quality of care. The lack of attention given to respectful, woman-centred care and over-medicalization is evident.
A41	Countries: Indonesia and Nigeria [43]	The objective is to analyse the patterns and factors influencing the utilisation of skilled birth attendance (SBA) services during childbirth in a country in Southern Asia (SA) and another country in Sub-Saharan Africa (SSA) over the past two decades.	A total of 63,924 participants were included in a cross-sectional study. Females aged 15 to 49	Intervention initiatives focused on improving health literacy and education among vulnerable populations, particularly rural communities and uneducated mothers, can greatly enhance health outcomes. Addressing regional disparities and shortages in healthcare and educational resources requires strong partnerships with local communities, the private sector, and all levels of government. These collaborations are crucial in expanding access to skilled birth attendants (SBAs) and strengthening maternal healthcare services.
A42	Countries Ethiopia [44]	This study aims to examine the quality of prenatal care and its related factors among pregnant women receiving care at government hospitals in the Sidama Region of Southern Ethiopia.	A random selection strategy was used to choose 72 pregnant women for a cross-sectional study.	Women with higher educational attainment tend to demand higher-quality care due to their greater awareness of available services, more positive perceptions of healthcare, and ability to identify key qualities in service providers. Additionally, increased education empowers individuals with greater autonomy in making informed healthcare decisions. Pregnant women who attended at least four antenatal care (ANC) visits at healthcare facilities and received guidance from medical professionals were more likely to access and benefit from high-quality medical interventions.
A43	Countries: Ghana and Tanzania [45]	This study aims to assess the initial level of routine antenatal and birth care in rural districts of Burkina Faso, Ghana, and Tanzania, as well as to identify any deficiencies or inadequacies.	A cross-sectional study was undertaken, encompassing a cohort of 63 postpartum women.	The findings reveal gaps in maternal healthcare services, including insufficient laboratory testing, inadequate counseling and health education programs, and poor monitoring and assessment of both the mother and infant during childbirth. Additionally, partographs were not utilized, and none of the assessed hospitals had forceps or vacuum extractors—both essential tools for assisted vaginal deliveries.

Each article was evaluated based on multiple criteria. A numerical score was assigned to each assessment item. Two researchers independently conducted the quality assessment, and any discrepancies were resolved through discussion or consultation with a third evaluator. Low-quality studies were not excluded; instead, they were considered in the overall analysis of study conclusions.

Given the expected diversity in study methodologies, populations, and outcomes, we adopted a narrative synthesis approach. A structured database was used to systematically categorize the extracted data and address the research objectives. Our analysis provides an overview of maternal and neonatal health outcomes and examines factors that facilitate or hinder the continuity of midwifery services in LMICs. Where feasible and appropriate, subgroup analyses may be conducted, considering variables such as geographic location, healthcare setting, and specific midwifery continuity of care models.

### Ethical considerations

Although this scoping review did not involve direct interaction with participants, ethical considerations remain important. The review utilized only data from previously published and publicly available studies. The reviewed studies complied with relevant ethical standards. All study data were obtained from legitimate sources and did not violate copyright or privacy.

### Registration of the study protocol

We preregistered the scoping review protocol in the Open Science Framework (OSF) before starting our analysis, helping to improve transparency and minimize potential bias. The protocol can be accessed through this link 10.17605/OSF.IO/8E3GM.

#### Findings

The scoping review aimed to provide insights into the present knowledge addressing midwifery continuity of care in LMICs. The review synthesized a large body of literature to accomplish this. A total of 43 research contributed to a deeper understanding of the implementation, outcomes, facilitators, and challenges related to midwifery continuity of care models in LMICs.

1. Characteristics of the Studies That Were Included

The publication time frame for the included studies ranged from 1980 to 2023, and they came from various LMICs in various parts of the world. The evaluation covered a wide range of research methodologies, including randomized controlled trials (RCTs), cohort studies, and qualitative investigations.

2. Establishment of a Continuity of Care Model for Midwifery

According to the review findings, LMICs have introduced midwifery continuity of care models to provide care centred on the woman and enhance the health of both mothers and newborns. These approaches entailed delegating comprehensive care provision during the prenatal, intrapartum, and postpartum periods to either a single known midwife or a group of midwives working together as a team. Here was a wide range of continuity, with some studies focusing on continuity of care during labour and deliveries and others extending continuity into the postpartum period [46].

The training and competency of midwives, collaboration with other healthcare professionals, integration into existing health systems, and community engagement were all necessary components to successfully implement midwifery continuity of care. Due to the adaptability of these models, local cultural norms and practices could be incorporated into the design [47]. However, several obstacles have been highlighted as hurdles to the plan’. These challenges include inadequate resources, inadequate infrastructure, and staff shortages. The most effective midwifery education is very important in providing high quality, life-saving services that can improve the health and well-being of mothers and babies [1].

3. Health Outcomes Regarding the Mother and the Newborn Child

An increasing body of evidence suggests that continuity of care provided by midwives has a positive influence on mother and newborn health outcomes in LMICs, as revealed by a synthesis of studies.

- A Lower Incidence of Medical Interventions: Several studies have found that women who receive midwifery continuity of care experience a lower incidence of medical interventions such as cesarean sections, instrumental births, and episiotomies. This decline was ascribed to the midwives’ increased focus on physiological birthing and other non-invasive labour and delivery techniques [48]
- Higher Rates of Physiological Birth: Continuity of care provided by midwives was associated with higher rates of physiological births, defined as vaginal deliveries that do not include any medical interventions. Women who received midwife care typically had shorter labour durations and fewer difficulties during childbirth [49]
- Maternal Satisfaction: Women who had continuity of care provided by a midwife reported higher overall satisfaction with their birthing experiences. The nurturing and individualized care provided by midwives contributed to a sense of control and empowerment for the client [50]
- Breastfeeding Initiation and Duration: Researchers found a correlation between midwifery continuity of care and higher rates of breastfeeding initiation and longer durations of exclusive breastfeeding. Providing breastfeeding education and assistance was an essential role that midwives performed [51]
- Neonatal Outcomes: Women who received midwifery continuity of care showed encouraging trends in neonatal outcomes, including lower newborn mortality and morbidity. This was one of the findings of the study. The individualized care and early postpartum assistance provided by midwives improved the health of newborns [52]

4. Factors That Help and Those That Hinder

Studies using qualitative methods shed light on the factors that make it easier and those that make it more challenging to implement midwifery continuity of care in LMICs.

- Facilitators: It was found that community acceptance of midwifery treatment, cultural competence on the part of midwives, and collaboration with traditional birth attendants were all factors that acted as facilitators. The capability of the midwives to negotiate the local norms and traditions was a contributing factor to the implementation’s overall success [53]
- Obstacles: The difficulties encountered were insufficient funding, a lack of governmental support, and opposition to change within healthcare systems. Obstacles were presented in the form of gender norms and societal expectations, which prevented the complete integration of midwifery care [13]

This scoping review highlights the growing evidence that supports the deployment of midwifery continuity of care models in LMICs. This analysis indicates that midwifery continuity of care models can improve maternal and neonatal health outcomes by reducing unnecessary interventions, promoting physiological birth, enhancing maternal satisfaction, supporting breastfeeding, and positively influencing neonatal well-being. The highlighted enablers and barriers give significant insights for policymakers and healthcare practitioners in LMICs interested in improving maternal and newborn care in LMICs through midwifery continuity of care. These findings contribute to the worldwide dialogue on improving mother and child health and underscore the need for individualized treatment focused on women in contexts when resources are limited [46].

## Discussion

To provide a more in-depth understanding of midwifery continuity of care in low and middle-income countries, the scoping review analyzed and summarized a vast amount of relevant research literature. Focusing on the significance of midwifery continuity of care in LMICs, the challenges encountered, and potential avenues for improving maternal and newborn health outcomes, this discussion section delves into the implications, limitations, and future directions that arise from the findings of this updated scoping review.

1. Importance of Midwifery Continuity of Care in Low- and Middle-Income Countries

The findings of this systematic study highlight the potential of continuity of care provided by midwives to address critical obstacles to maternal and neonatal health in LMICs. Midwifery continuity of care provides a woman-centred, holistic approach that aligns with the ideals of respectful and culturally sensitive care [45]. This method is especially beneficial in contexts where resources are frequently restricted. The global attempt to limit the needless amount of medical intervention during childbirth fits with the focus on physiological birth and fewer interventions by medical professionals. The positive outcomes observed regarding decreased interventions, increased rates of physiological birth, improved maternal satisfaction, and enhanced breastfeeding initiation and duration suggest that midwifery continuity of care holds promise in improving maternal and newborn health outcomes in LMICs [42].

2. Challenges to Overcome and Adaptations

In LMICs, the assessment brought to light several obstacles that must be overcome before midwifery continuity of care can be implemented. Various obstacles, including insufficient resources and infrastructure, a lack of qualified workers, and labour shortages frequently hinder the scalability of midwifery programs. It is possible for socio-cultural factors, such as gender norms and traditional practices, to influence community acceptability of midwifery services and utilization. In addition, there is a lack of governmental backing and opposition to change within healthcare systems, both of which create substantial challenges [28, 29, 41].

Nevertheless, one of the most prominent themes that surfaced was the adaptability and flexibility of the midwifery continuity of care approaches. The ability to adapt these models to the specifics of different cultural settings and contexts is crucial to effective implemention of these models. The possibility of integrating midwifery care within already established health systems is illustrated through projects that involve collaborative efforts between midwives, traditional birth attendants, and other healthcare practitioners. The robustness of midwifery continuity of care approaches is demonstrated by these adjustments, as is their capacity to address the particular issues presented by LMIC settings [14].

Another study highlighted the absence of midwifery continuity of care initiatives in LMICs and emphasized the need for greater investment in establishing effective midwifery systems [46] This should be complemented by ongoing monitoring, evaluation, and research to assess the impact, benefits, and challenges of different models across various contexts. Operational research is essential to identify the barriers, enablers, and constraints affecting the implementation of midwifery continuity of care models, particularly in regions facing midwife shortages. Strengthening midwifery education and regulatory frameworks, along with fostering a supportive healthcare environment, is crucial for ensuring a smooth transition and sustained continuity of midwifery care in LMICs [54–56]. The Village Midwife Program in Indonesia exemplifies the successful integration of midwifery services into the national healthcare system by deploying midwives in rural communities to enhance maternal and neonatal healthcare access, supported by continuous training and community partnership [57, 58] This program secured long-term funding through a combination of government budget al.locations and international donor contributions, such as from the World Bank, to support the construction of maternal and child health clinics and training centers [59] Similarly, Nepal’s Skilled Birth Attendant (SBA) initiative has trained over 7,000 midwifery professionals since 2003, significantly reducing maternal mortality rates through competency-based training and strong community engagement [60] Nigeria’s Midwives Service Scheme (MSS) has demonstrated an effective approach in integrating midwifery into rural healthcare by stationing midwives in primary health centers and strengthening referral systems, although challenges in sustainable funding remain [61] In terms of training expansion and strengthening community partnerships, the UNFPA and ICM initiative in Asia has aligned midwifery education standards with national policies, engaging over 60 participants from seven countries to enhance training programs and broaden collaboration with donor organizations and key stakeholders [62, 63] The ability to attract sustained funding in this initiative was largely due to multi-sectoral collaboration, where private sector donors and international development agencies provided financial backing while governments committed to co-financing midwifery education and service integration [64].

3. Public Policy and Professional Practice

The findings of this evidence synthesis have significant ramifications for public policy and clinical practice in LMICs. Policymakers and healthcare administrators should consider integrating midwifery continuity of care models into existing maternal and newborn healthcare systems. For midwifery care programs to be successfully implemented and maintained over time, it is vital to develop strategies to overcome the obstacles that have been identified. These strategies include raising financing, adopting supportive regulations, and fighting for gender equity [14].

Healthcare practitioners, including midwives, play the most crucial role in advocacy and provision of midwifery continuity of care. Therefore, midwives should have unhinged access to comprehensive training programs that emphasize cultural competence, communication skills, and collaboration which will empower them to provide highest quality of care in diverse LMIC environments. Midwifery services can experience significant improvements in both quality and effect if ongoing professional development and capacity-building efforts are implemented [5].

4. Future Research

This scoping review offers valuable insights while highlighting key areas that need further exploration. One promising direction is investigating how digital health technologies can improve midwifery care and address maternal and neonatal health inequities. International health organizations also emphasize the importance of enhancing midwives’ skills to ensure high-quality services for mothers and newborns [65].

Further studies should also explore the cost-effectiveness of midwifery continuity of care models in LMICs. Economic evaluations comparing these models with existing healthcare systems can offer policymakers critical insights to guide resource allocation and decision-making [6] Also, investigating the long-term impact of midwifery continuity of care on maternal and child health beyond the immediate postpartum period is essential.

Another key research priority is exploring how cultural and socioeconomic factors shape the acceptance and implementation of midwifery continuity of care. Qualitative studies examining traditional beliefs, community perspectives, and sociocultural dynamics can inform the development of culturally responsive midwifery care models tailored to specific regional needs. This approach is vital for ensuring effective implementation, sustainability, and long-term success [27].

### Limitations of the study

This study has several limitations. First, the reliance on studies published in English may introduce language bias, potentially excluding relevant research in other languages. Also, the inclusion of studies spanning a long timeframe (1932–2023) may result in data heterogeneity due to evolving healthcare systems, policies, and practices over time.

Despite these limitations, this study provides valuable insights into the impact of midwifery continuity of care on maternal and neonatal outcomes in low- and middle-income countries (LMICs). Furthermore, it comprehensively discusses the challenges associated with implementing these models and explores potential strategies to address them.

## Conclusion

The review highlights how midwifery continuity of care can transform maternal and newborn health in LMICs. When mothers receive consistent, compassionate care throughout pregnancy, childbirth, and postpartum, their health outcomes improve significantly. This model also supports Sustainable Development Goal (SDG) 3.2, which focuses on reducing preventable newborn and child deaths by 2030.

Midwifery continuity of care is more than a healthcare model. It is a commitment to trust, dignity, and ensuring that every mother and baby, regardless of where they live, receive the best possible start in life. By addressing the real challenges in resource-limited settings, it not only improves health outcomes but also fosters equity, giving women the power to make informed choices about their care.

Its impact extends far beyond the birth room. With a proven ability to reduce unnecessary medical interventions and support physiological birthing practices, midwifery continuity of care stands as a cornerstone of future maternal and newborn health strategies. Its resilience and adaptability make it a vital approach for strengthening health systems, particularly in low- and middle-income countries, where the burden of maternal and newborn mortality remains high.

To realize its full potential, policymakers, healthcare practitioners, and researchers must prioritize investments in midwifery-led care. Scaling up these models, integrating them into existing health systems, and expanding training opportunities will not only improve maternal and newborn health outcomes but also contribute to broader efforts toward equitable health care in the future.

## Supplementary Information

Supplementary Material 1.

## Data Availability

All pertinent materials and data that support the findings of this review are included within the manuscript.

## Abbreviations

ANC: Antenatal Care
CE: Community Engagement
CHWs: Community Health Workers
EmOC: Emergency Obstetric Care
ENC: Essential Newborn Care
HRH: Human Resources for Health
JBI: Joanna Briggs Institute
LMICs: Low- and Middle-Income Countries
mHealth: Mobile Health
MNH: Maternal and Newborn Health
OCN: Obstetric Care Navigation
RCTs: Randomized Controlled Trials
SBA: Skilled Birth Attendant
SDGs: Sustainable Development Goals
TBA: Traditional Birth Attendant
WHO: World Health Organization

## References

[1] Adatara P, Amooba PA, Afaya A, Salia SM, Avane MA, Kuug A et al. Challenges experienced by midwives working in rural communities in the upper East region of Ghana: a qualitative study. BMC Pregnancy Childbirth. 2021;21(1). 10.1186/s12884-021-03762-0 PMC8033657 33836689

[2] Shakespeare C, Merriel A, Bakhbakhi D, Baneszova R, Barnard K, Lynch M, et al. Parents’ and healthcare professionals’ experiences of care after stillbirth in low- and middle‐income countries: a systematic review and meta‐summary. BJOG. 2019;126(1):12–21. 30099831 10.1111/1471-0528.15430

[3] Mangham-Jefferies L, Pitt C, Cousens S, Mills A, Schellenberg J. Cost-effectiveness of strategies to improve the utilization and provision of maternal and newborn health care in low-income and lower-middle-income countries: A systematic review. BMC Pregnancy Childbirth. 2014;14(1). 10.1186/1471-2393-14-243 PMC4223592 25052536

[4] Arksey H, O’Malley L. Scoping studies: towards a methodological framework. Int J Social Res Methodology: Theory Pract. 2005;8(1):19–32.

[5] Aregay A, Oconnor M, Stow J, Ayers N, Lee S. Strategies used to Establish palliative care in rural low-and middle-income countries: an integrative review. Health Policy Plan. 2020;35(8):1110–29. 10.1093/heapol/czaa051 32577766

[6] Austad K, Juarez M, Shryer H, Moratoya C, Rohloff P. Obstetric care navigation: results of a quality improvement project to provide accompaniment to women for facility-based maternity care in rural Guatemala. BMJ Qual Saf. 2020;29(2):169–78. 31678958 10.1136/bmjqs-2019-009524 PMC7045784

[7] Bartlett L, Weissman E, Gubin R, Patton-Molitors R, Friberg IK. The impact and cost of scaling up midwifery and obstetrics in 58 low- and middle-income countries. PLoS ONE. 2014;9(6). 10.1371/journal.pone.0098550 PMC4062394 24941336

[8] Bolan N, Cowgill KD, Walker K, Kak L, Shaver T, Moxon S, et al. Human resources for Health-Related challenges to ensuring quality newborn care in Low-and Middle-Income countries: A scoping review. Global Health Sci Pract. 2021;9(1):160–76 Available from: www.ghspjournal.org. 10.9745/GHSP-D-20-00362 PMC8087437 33795367

[9] Carlo WA, McClure EM, Chomba E, Chakraborty H, Hartwell T, Harris H, et al. Newborn care training of midwives and neonatal and perinatal mortality rates in a developing country. Pediatrics. 2010;126(5):e1064–71. 20937659 10.1542/peds.2009-3464

[10] Chowdhury ME, RC, KJ AI, GK, DGS. Equity in use of home-based or facility-based skilled obstetric care in rural Bangladesh: an observational study. Lancet. 2006;367(9507):327–32. 16443040 10.1016/S0140-6736(06)68070-7

[11] Dada S, De Brún A, Banda EN, Bhattacharya S, Mutunga Z, Gilmore B. A realist review protocol on communications for community engagement in maternal and newborn health programmes in low- and middle-income countries. Syst Rev. 2022;11(1). 10.1186/s13643-022-02061-9 PMC9465973 36096841

[12] Dowswell T, Carroli G, Duley L, Gates S, Gülmezoglu AM, Khan-Neelofur D, et al. Alternative versus standard packages of antenatal care for low-risk pregnancy. Cochrane Database Syst Rev. 2015;2015(7):CD000934. 10.1002/14651858.CD000934. 10.1002/14651858.CD000934.pub3 PMC7061257 26184394

[13] Filby A, McConville F, Portela A. What prevents quality midwifery care? A systematic mapping of barriers in low and middle income countries from the provider perspective. PLoS ONE. 2016;11(5). 10.1371/journal.pone.0153391 PMC4852911 27135248

[14] Garces A, McClure EM, Espinoza L, Saleem S, Figueroa L, Bucher S, et al. Traditional birth attendants and birth outcomes in low-middle income countries: A review. Semin Perinatol. 2019;43(5):247-51. 10.1053/j.semperi.2019.03.013 PMC6591059 30981470

[15] Gathara D, Zosi M, Serem G, Nzinga J, Murphy GAV, Jackson D, et al. Developing metrics for nursing quality of care for low- and middle-income countries: A scoping review linked to stakeholder engagement. Hum Resour Health. 2020;18:34. 10.1186/s12960-020-00470-2 PMC7222310 32410633

[16] Gavine A, MacGillivray S, McConville F, Gandhi M, Renfrew MJ. Pre-service and in-service education and training for maternal and newborn care providers in low- and middle-income countries: an evidence review and gap analysis. Midwifery. 2019;78:104–13. 31419781 10.1016/j.midw.2019.08.007

[17] Goldman N, Glei DA. Evaluation of midwifery care: results from a survey in rural Guatemala. Soc Sci Med. 2003;56(4):685–700. 10.1016/s0277-9536(02)00065-5 12560004

[18] Golladan FL. Community health care in developing countries. Finance Dev. 1980;17(3):35–9. 12262079

[19] Grady K, Ameh C, Adegoke A, Kongnyuy E, Dornan J, Falconer T, et al. Improving essential obstetric and newborn care in resource-poor countries. J Obstet Gynaecol (Lahore). 2011;31(1):18–23. 10.3109/01443615.2010.533218 21280987

[20] Harrison KA. Maternal mortality in developing countries. BJOG. 1989;96(1):1–3. 10.1111/j.1471-0528.1989.tb01567.x 2647128

[21] Hundley VA, Avan BI, Braunholtz D, Fitzmaurice AE, Graham WJ. Lessons regarding the use of birth kits in low resource countries. Midwifery. 2011;27(6). 10.1016/j.midw.2010.10.003 21051126

[22] Ismaila Y, Bayes S, Geraghty S. Midwives’ strategies for coping with barriers to providing quality maternal and neonatal care: a glaserian grounded theory study. BMC Health Serv Res. 2021;21(1). 10.1186/s12913-021-07049-0 PMC8565049 34732179

[23] Joseph G, Da Silva ICM, Wehrmeister FC, Barros AJD, Victora CG. Inequalities in the coverage of place of delivery and skilled birth attendance: analyses of cross-sectional surveys in 80 low and middle-income countries. Reprod Health. 2016;13(1). 10.1186/s12978-016-0192-2 PMC4912761 27316970

[24] Kurji Z, Premani ZS, Mithani Y. Review and analysis of quality healthcare system enhancement in developing countries. J Pak Med Assoc. 2015;65(7):776–81. 26160090

[25] Ladur AN, van Teijlingen E, Hundley V. Male involvement in promotion of safe motherhood in low- and middle-income countries: A scoping review. Midwifery. 2021;103:103089. 34293604 10.1016/j.midw.2021.103089

[26] Metcalfe R, Adegoke AA. Strategies to increase facility-based skilled birth attendance in South Asia: a literature review. Int Health. 2013;5:96–105. 24030109 10.1093/inthealth/ihs001

[27] Michel-Schuldt M, McFadden A, Renfrew M, Homer C. The provision of midwife-led care in low-and middle-income countries: an integrative review. Midwifery. 2020;84:102659. 10.1016/j.midw.2020.102659 32062187

[28] Mohseni M, Mousavi Isfahani H, Moosavi A, Dehghanpour Mohammadian E, Mirmohammadi F, Ghazanfari F, et al. Health system-related barriers to prenatal care management in low- and middle-income countries: A systematic review of the qualitative literature. Prim Health Care Res Dev. 2023;24:e15. 10.1017/S1463423622000706 PMC9972358 36843095

[29] Munabi-Babigumira S, Glenton C, Lewin S, Fretheim A, Nabudere H. Factors that influence the provision of intrapartum and postnatal care by skilled birth attendants in low- and middle-income countries: a qualitative evidence synthesis. Cochrane Database Syst Rev. 2017;2018:2. 10.1002/14651858.CD011558.pub2 PMC5721625 29148566

[30] Nove A, Bazirete O, Hughes K, Turkmani S, Callander E, Scarf V, et al. Which low- and middle-income countries have midwife-led birthing centres and what are the main characteristics of these centres? A scoping review and scoping survey. Midwifery. 2023;123:103717. 10.1016/j.midw.2023.103717 PMC10281083 37182478

[31] Peven K, Bick D, Purssell E, Rotevatn TA, Nielsen JH, Taylor C. Evaluating implementation strategies for essential newborn care interventions in low- and low middle-income countries: A systematic review. Health Policy Plan. 2021;35(Supplement 2):ii47-65. 10.1093/heapol/czaa122 PMC7646733 33156939

[32] Pyone T, Sorensen BL, Tellier S. Childbirth attendance strategies and their impact on maternal mortality and morbidity in low-income settings: A systematic review. Acta Obstet Gynecol Scand. 2012;91:1029–37. 10.1111/j.1600-0412.2012.01460.x 22583081

[33] Sangy MT, Duaso M, Feeley C, Walker S. Barriers and facilitators to the implementation of midwife-led care for childbearing women in low- and middle-income countries: A mixed-methods systematic review. Midwifery. 2023;122:103696. 10.1016/j.midw.2023.103696 37099826

[34] Schwerdtle P, Morphet J, Hall H. A scoping review of mentorship of health personnel to improve the quality of health care in low and middle-income countries. Global Health. 2017;13(1). 10.1186/s12992-017-0301-1 PMC5627414 28974233

[35] Sibley L, Armbruster D. Obstetric first aid in the community-partners in safe motherhood a Strategy for Reducing Maternal Mortality. J Nurse Midwifery. 1997;42(2):117–21. 9107120 10.1016/s0091-2182(97)00022-0

[36] Singh PK, Singh L. Examining inter-generational differentials in maternal health care service utilization: insights from the Indian demographic and health survey. J Biosoc Sci. 2014;46(3):366–85. 23866261 10.1017/S0021932013000370

[37] Stansert Katzen L, Dippenaar E, Laurenzi CA, Rotheram Borus MJ, le Roux K, Skeen S, et al. Community health workers’ experiences of supervision in maternal and child health programmes in low- and middle-income countries: A qualitative evidence synthesis. Health and Social Care in the Community, vol. 30. John Wiley and Sons Inc; 2022. pp. 2170–85. 10.1111/hsc.13893 35852501

[38] Stewart L, Usher K. The impact of nursing leadership on patient safety in a developing country. J Clin Nurs. 2010;19(21–22):3152–60. 21040019 10.1111/j.1365-2702.2010.03285.x

[39] Suryanto PV, Boyle M. Healthcare system in Indonesia. Hosp Top. 2017;95(4):82–9. 28636456 10.1080/00185868.2017.1333806

[40] Trevisanuto D, Bavuusuren B, Wickramasinghe CS, Dharmaratne SM, Doglioni N, Giordan A, et al. Improving maternal and neonatal departments in high and low resource settings: the opinion of local health providers. J Maternal-Fetal Neonatal Med. 2011;24(10):1267–72. 10.3109/14767058.2010.546911 21261448

[41] Ueno E, Adegoke AA, Masenga G, Fimbo J, Msuya SE. Skilled birth attendants in Tanzania: A descriptive study of cadres and emergency obstetric care signal functions performed. Matern Child Health J. 2015;19(1):155–69. 24791974 10.1007/s10995-014-1506-z

[42] Van Lerberghe W, Matthews Z, Achadi E, Ancona C, Campbell J, Channon A, et al. Country experience with strengthening of health systems and deployment of midwives in countries with high maternal mortality. Lancet. 2014;384(9949):1215–25. 10.1016/S0140-6736(14)60919-3 24965819

[43] Walker T, Woldegiorgis M, Bhowmik J. Utilisation of skilled birth attendant in low-and middle-income countries: trajectories and key sociodemographic factors. Int J Environ Res Public Health. 2021;18(20). 10.3390/ijerph182010722 PMC8535845 34682468

[44] Kare AP, Gujo AB, Yote NY. Quality of antenatal care and associated factors among pregnant women attending government hospitals in Sidama region, Southern Ethiopia. SAGE Open Med. 2021;9:20503121211058055. 10.1177/20503121211058055 PMC8640313 34868590

[45] Duysburgh E, Zhang WH, Ye M, Williams A, Massawe S, Sié A, et al. Quality of antenatal and childbirth care in selected rural health facilities in Burkina Faso, Ghana and Tanzania: similar finding. Trop Med Int Health. 2013;18(5):534–47. 23398053 10.1111/tmi.12076

[46] Bradford BF, Wilson AN, Portela A, McConville F, Turienzo CF, Homer CSE. Midwifery continuity of care: A scoping review of where, how, by whom and for whom? PLOS Glob Public Health. 2022;2(10):e0000935. 10.1371/journal.pgph.0000935 PMC10021789 36962588

[47] Hanafin S. Considerations in integrating hospital-based midwifery services into the community. Br J Commun Nurs. 2017;22. Available from: www.bjcn.co.uk 10.12968/bjcn.2017.22.2.92 28161973

[48] Sandall J, Fernandez Turienzo C, Devane D, Soltani H, Gillespie P, Gates S, et al. Midwife continuity of care models versus other models of care for childbearing women. Cochrane Database Syst Rev. 2024;4(4):CD004667. 10.1002/14651858.CD004667.pub6 PMC11005019 38597126

[49] Lundborg L, Åberg K, Liu X, Norman M, Stephansson O, Pettersson K, et al. Midwifery Continuity of Care During Pregnancy, Birth, and the Postpartum Period: A Matched Cohort Study. Birth. 2024. 10.1111/birt.12875 PMC11829270 39465909

[50] Shahinfar S, Abedi P, Najafian M, Abbaspoor Z, Mohammadi E, Alianmoghaddam N. Women’s perception of continuity of team midwifery care in Iran: a qualitative content analysis. BMC Pregnancy Childbirth. 2021;21(1). 10.1186/s12884-021-03666-z PMC7922712 33653289

[51] Dagla M, Mrvoljak-Theodoropoulou I, Vogiatzoglou M, Giamalidou A, Tsolaridou E, Mavrou M, et al. Association between breastfeeding duration and long-term midwifery-led support and psychosocial support: outcomes from a Greek non-randomized controlled perinatal health intervention. Int J Environ Res Public Health. 2021;18(4):1–18. 10.3390/ijerph18041988 PMC7922856 33670797

[52] Mortensen B, Lieng M, Diep LM, Lukasse M, Atieh K, Fosse E. Improving maternal and neonatal health by a Midwife-Led continuity model of Care– An observational study in one governmental hospital in Palestine. EClinicalMedicine. 2019;10:84–91. 31193799 10.1016/j.eclinm.2019.04.003 PMC6543174

[53] Musie MR, Mulaudzi MF, Anokwuru R, Bhana-Pema V. Recognise and acknowledge Us: views of traditional birth attendants on collaboration with midwives for maternal health care services. Int J Reprod Med. 2022;2022:1–10. 10.1155/2022/9216500 PMC9300345 35874464

[54] Liu Y, Li T, Guo N, Jiang H, Li Y, Xu C et al. Women’s experience and satisfaction with midwife-led maternity care: a cross-sectional survey in China. BMC Pregnancy Childbirth. 2021;21(1). 10.1186/s12884-021-03638-3 PMC7893951 33607963

[55] Thommesen T, Kismul H, Kaplan I, Safi K, Van Den Bergh G. The midwife helped me. otherwise i could have died: women’s experience of professional midwifery services in rural Afghanistan - A qualitative study in the provinces Kunar and Laghman. BMC Pregnancy Childbirth. 2020;20(1). 10.1186/s12884-020-2818-1 PMC7059669 32138695

[56] WHO. Kesehatan Neonatal Rencana Aksi Nasional. 2014.

[57] WHO. The Republic of Indonesia health system review Asia Pacific observatory on health systems and policies. Health Systems in Transition. 2017;7(1).

[58] UNFPA. Laporan Konsultansi Kebidanan. 2014.

[59] BKKBN KKBUI dan KBK. Strategi Pelaksanaan Program Keluarga Berencana Berbasis Hak untuk Percepatan Akses ke Pelayanan Keluarga Berencana dan Kesehatan Reproduksi yang Terintegrasi dalam Mencapai Tujuan Pembangunan Indonesia. UNFPA; 2018.

[60] Aryal S, Nepal S. An insight into two decades of skilled birth attendants in Nepal. Front Glob Womens Health. 2022;3:899010. 10.3389/fgwh.2022.899010 PMC9411965 36033921

[61] UNFPA. Building a health workforce to meet the needs of women, newborns and adolescents everywhere The STATE OF THE World’s Midwifery. 2021.

[62] UNFPA. Landmark Action on Midwifery. 2011.

[63] ICM. International Confederation of Midwives Annual Report 2018. International Confederation of Midwives; 2018.

[64] UNFPA. Leaving No One Behind in a Global Crisis through Universal Access to Sexual and Reproductive Health Services and Information. 2022.

[65] Nove A, Friberg IK, de Bernis L, McConville F, Moran AC, Najjemba M, et al. Potential impact of midwives in preventing and reducing maternal and neonatal mortality and stillbirths: a lives saved tool modelling study. Lancet Glob Health. 2021;9(1):e24–32. 33275948 10.1016/S2214-109X(20)30397-1 PMC7758876

