# Supplementary material for: Ninety-one years of midwifery continuity of care in low and middle-income countries: a scoping review
Source: BMC Health Serv Res. 2025 Mar 28;25:463. doi: 10.1186/s12913-025-12612-0 (PMC11951775; doi:10.1186/s12913-025-12612-0)
Supplement: Supplementary file 1 — Supplementary Material 1. [file 12913_2025_12612_MOESM1_ESM.docx]

Table 3 JBI Critical Appraisal Checklist for systematic reviews and research syntheses for 20 included studies

| **Question** | **A1** | **A4** | **A7** | **A9** | **A10** | **A11** | **A12** | **A17** | **A20** | **A21** | **A22** | **A23** | **A24** | **A25** | **A27** | **A28** | **A29** | **A30** | **A31** | **A32** |
| --- | --- | --- | --- | --- | --- | --- | --- | --- | --- | --- | --- | --- | --- | --- | --- | --- | --- | --- | --- | --- |
| Is the review question stated in an explicit and straightforward manner? | **3** | **3** | **3** | **3** | **3** | **3** | **3** | **3** | **3** | **3** | **3** | **3** | **3** | **3** | **3** | **3** | **3** | **3** | **3** | **3** |
| The review question and the inclusion criteria: were they appropriate? | **3** | **3** | **3** | **3** | **3** | **3** | **3** | **3** | **3** | **3** | **3** | **3** | **3** | **3** | **3** | **3** | **3** | **3** | **3** | **3** |
| Was the search plan appropriate? | **3** | **3** | **3** | **3** | **3** | **3** | **3** | **3** | **3** | **3** | **3** | **3** | **3** | **3** | **3** | **3** | **3** | **3** | **3** | **3** |
| Were the materials and sources used to look for studies sufficient? | **3** | **3** | **3** | **3** | **3** | **3** | **3** | **3** | **3** | **3** | **3** | **3** | **3** | **3** | **3** | **3** | **3** | **3** | **3** | **3** |
| Were the evaluation standards for the studies suitable? | **3** | **3** | **3** | **3** | **3** | **3** | **3** | **3** | **3** | **3** | **3** | **3** | **3** | **3** | **3** | **3** | **3** | **3** | **3** | **3** |
| Was the critical evaluation carried out independently by two or more reviewers? | **1** | **1** | **3** | **3** | **1** | **3** | **3** | **3** | **1** | **3** | **3** | **1** | **3** | **3** | **3** | **3** | **3** | **3** | **3** | **3** |
| Were there techniques to reduce data extraction errors? | **3** | **3** | **3** | **3** | **3** | **3** | **3** | **3** | **3** | **3** | **3** | **3** | **3** | **3** | **3** | **3** | **3** | **3** | **3** | **3** |
| Were the techniques employed to integrate the studies suitable? | **3** | **3** | **3** | **3** | **3** | **3** | **3** | **3** | **3** | **3** | **3** | **3** | **3** | **3** | **3** | **3** | **3** | **3** | **3** | **3** |
| Has the risk of publication bias been evaluated? | **2** | **2** | **2** | **2** | **2** | **2** | **2** | **2** | **2** | **2** | **2** | **2** | **2** | **2** | **2** | **2** | **2** | **2** | **2** | **2** |
| Were the presented results consistent with suggestions for policy and/or practice? | **3** | **3** | **3** | **3** | **3** | **3** | **3** | **3** | **3** | **3** | **3** | **3** | **3** | **3** | **3** | **3** | **3** | **3** | **3** | **3** |
| Were the particular guidelines for fresh research suitable? | **3** | **3** | **3** | **3** | **3** | **3** | **3** | **3** | **3** | **3** | **3** | **3** | **3** | **3** | **3** | **3** | **3** | **3** | **3** | **3** |
| Total | **30** | **30** | **32** | **32** | **30** | **32** | **32** | **32** | **30** | **32** | **32** | **30** | **32** | **32** | **32** | **32** | **32** | **32** | **32** | **32** |

Source: JBI Critical Appraisal Tools

Table 4 JBI Critical Appraisal Checklist for RCTs to 2 included studies

| **Question** | **A8** | **A15** |
| --- | --- | --- |
| Is it true randomization that was used to assign participants to treatment groups? | **3** | **3** |
| Was the assignment to treatment groups hidden? | **3** | **2** |
| At baseline, were treatment groups comparable? | **3** | **3** |
| Did individuals lack awareness of their assigned treatment? | **2** | **1** |
| Were those administering the treatment unaware of the assignment? | **2** | **1** |
| Did outcome assessors lack awareness of the treatment assignment? | **2** | **1** |
| With the exception of the relevant intervention, were treatment groups treated in the same way? | **3** | **3** |
| Was follow-up done to the end, and if not, were the variations in follow-up between the groups sufficiently explained and examined? | **3** | **3** |
| In the groups to which they were assigned at random, were the participants examined? | **3** | **3** |
| Did treatment groups' results follow the same metrics? | **3** | **3** |
| Were results measured in an accurate manner? | **3** | **3** |
| Was the right statistical analysis applied? | **3** | **3** |
| Did the study's conduct and analysis take into consideration any modifications from the conventional RCT design, such as individual randomization and parallel groups, and was the trial design appropriate? | **3** | **3** |
| **Total** | **36** | **32** |

Source: JBI Critical Appraisal Tools

Table 5. JBI Critical Appraisal Checklist for analytical cross sectional studies for 9 included studies

| **Question** | **A3** | **A6** | **A16** | **A19** | **A34** | **A39** | **A41** | **A42** | **A43** |
| --- | --- | --- | --- | --- | --- | --- | --- | --- | --- |
| Were the requirements for sample inclusion well-defined? | 3 | 3 | 3 | 3 | 3 | 3 | 3 | 3 | 3 |
| Were the study participants and the environment thoroughly described? | 3 | 3 | 3 | 3 | 3 | 3 | 3 | 3 | 3 |
| Was the exposure measured in a method that was accurate and valid? | 3 | 3 | 3 | 3 | 3 | 3 | 3 | 3 | 3 |
| Were impartial, accepted standards employed to measure the condition? | 3 | 3 | 3 | 3 | 3 | 3 | 3 | 3 | 3 |
| Were confounding variables found? | 3 | 3 | 3 | 3 | 3 | 3 | 3 | 3 | 3 |
| Were confounding factor strategies mentioned? | 3 | 3 | 2 | 3 | 3 | 3 | 3 | 3 | 2 |
| Were the results assessed using a valid and trustworthy method? | 3 | 3 | 3 | 3 | 3 | 3 | 3 | 3 | 3 |
| Was the right statistical analysis applied? | 3 | 3 | 3 | 3 | 3 | 3 | 3 | 3 | 3 |
| **Total** | **24** | **24** | **23** | **24** | **24** | **24** | **24** | **24** | **23** |

Source: JBI Critical Appraisal Tools

Table 6. JBI Critical Appraisal Checklist for Qualitative Research for eight included studies

| **Question** | **13** | **14** | **18** | **26** | **35** | **36** | **38** | **40** |
| --- | --- | --- | --- | --- | --- | --- | --- | --- |
| Do the study methodology and the declared philosophical perspective align? | 3 | 3 | 3 | 3 | 3 | 3 | 3 | 3 |
| Do the research question and objectives align with the research methodology? | 3 | 3 | 3 | 3 | 3 | 3 | 3 | 3 |
| Are the data collection techniques and the research approach in line with each other? | 3 | 3 | 3 | 3 | 3 | 3 | 3 | 3 |
| Are the data analysis and representation, as well as the study technique, consistent with each other? | 3 | 3 | 3 | 3 | 3 | 3 | 3 | 3 |
| Are the methods used in the research and the way the findings are interpreted consistent? | 3 | 3 | 3 | 3 | 3 | 3 | 3 | 3 |
| Exists a remark that places the researcher in a theoretical or cultural context? | 2 | 2 | 3 | 2 | 3 | 3 | 2 | 2 |
| Is the researcher's impact on the research and vice versa taken into consideration? | 2 | 2 | 3 | 2 | 3 | 3 | 2 | 2 |
| Are the voices and the participants appropriately represented? | 3 | 3 | 3 | 3 | 3 | 3 | 3 | 3 |
| Is there evidence of ethical approval from a suitable body, or, in the case of recent studies, is the research ethical in accordance with current standards? | 3 | 3 | 3 | 3 | 3 | 3 | 3 | 3 |
| Do the research report's conclusions stem from the data's analysis or interpretation? | 3 | 3 | 3 | 3 | 3 | 3 | 3 | 3 |
| **Total** | **28** | **28** | **30** | **28** | **30** | **30** | **28** | **28** |

Source: JBI Critical Appraisal Tools

Table 7. JBI Critical Appraisal Checklist for cohort studies for four included studies

| **Question** | **A2** | **A5** | **A33** | **A37** |
| --- | --- | --- | --- | --- |
| Did the two groups come from the same population and share similarities? | 3 | 3 | 3 | 3 |
| Did individuals in the exposed and unexposed groups receive identical measurements of their exposures? | 3 | 3 | 3 | 3 |
| Was the exposure measured in a method that was accurate and valid? | 3 | 3 | 3 | 3 |
| Were confounding variables found? |  | 3 | 3 | 3 |
| Were confounding factor strategies mentioned? | 33 | 3 | 3 | 3 |
| At the beginning of the study (or at the time of exposure), were the groups/participants free of the outcome? | 3 | 3 | 3 | 3 |
| Were the results assessed using a valid and trustworthy method? | 3 | 3 | 3 | 3 |
| Was the follow-up period adequately documented and of sufficient duration to allow for the occurrence of the outcomes?  Top of Form  Bottom of Form | 3 | 3 | 3 | 3 |
| Was the follow-up conducted to the end, and if not, were the reasons why it was discontinued explained and investigated? | 3 | 3 | 2 | 2 |
| Were techniques used to deal with inadequate follow-up? | 3 | 3 | 2 | 2 |
| Was the right statistical analysis applied? | 3 | 3 | 3 | 3 |
| **Total** | **33** | **33** | **31** | **31** |

Source: JBI Critical Appraisal Tools

Notes: JBI Ceklist Using Numerical Scores

Score of 0=not applicable, 1=Unclear, 2=No, and 3=Yes

Methodological Quality Assessment Using the JBI Appraisal Tool

In evaluating the studies included in this systematic review, we utilized the Joanna Briggs Institute (JBI) Critical Appraisal Checklists to assess the methodological quality across different study designs. The appraisal covered various types of studies, including systematic reviews, randomized controlled trials (RCTs), cohort studies, cross-sectional studies, and qualitative research. This comprehensive assessment was essential to ensure the reliability and validity of the conclusions drawn from the reviewed literature.

The JBI Critical Appraisal Checklist for Systematic Reviews and Research Syntheses revealed that all included reviews had clearly stated research questions and appropriate inclusion criteria. The search strategies and sources used were adequate, and methods for appraising studies were appropriately applied. Most reviews had recommendations supported by the reported data and specific directives for future research, with scores generally ranging from 30 to 32 out of a possible 32 points, indicating high methodological quality.

For RCTs, the JBI checklist indicated that studies employed true randomization and appropriate statistical analyses. However, there were some limitations regarding blinding of participants and outcome assessors, with scores of 32 and 36 out of 39. Cohort studies showed strong adherence to the criteria, with scores of 31 to 33 out of 33, highlighting robust methodological approaches, particularly in the measurement of exposures and outcomes.

Cross-sectional studies were assessed using the JBI Checklist, and the studies generally met the criteria for inclusion, description of subjects and settings, and the use of valid and reliable measurement methods. Confounding factors were identified and strategies to address them were stated, although some studies had minor limitations in this area. The scores for these studies were consistently high, ranging from 23 to 24 out of 24.

Qualitative studies were appraised for congruity between the stated philosophical perspective, methodology, data collection methods, and interpretation of results. Most qualitative studies achieved high scores (28 to 30 out of 30), reflecting strong methodological congruence and ethical considerations.

In summary, the JBI appraisal indicated that the majority of studies included in this review were of high methodological quality, providing a strong evidence base for the conclusions drawn. This comprehensive evaluation enhances the credibility of the findings related to midwifery continuity of care in low- and middle-income countries (LMICs), emphasizing the positive impacts on maternal and neonatal health outcomes.
